# Genetic association of *FTO* gene polymorphisms with obesity and its related phenotypes: A case-control study

**DOI:** 10.34172/jcvtr.33038

**Published:** 2024-06-25

**Authors:** Tanmayi Sharma, Badaruddoza Badaruddoza

**Affiliations:** Department of Human Genetics, Guru Nanak Dev University, Amritsar-143 005, Punjab, India

**Keywords:** FTO gene, Haplotype, Linkage disequilibrium, Obesity, Sanger sequencing

## Abstract

**Introduction:**

*FTO* gene belongs to the non-heme Fe (II) and 2 oxoglutarate-dependent dioxygenase superfamily. Polymorphisms within the first intron of the *FTO* gene have been examined across various populations, yielding disparate findings.The present study aimed to determine the impact of two intronic polymorphisms *FTO* 30685T/G (rs17817449) and -23525T/A (rs9939609) on the risk of obesity in Punjab, India.

**Methods:**

Genotypic and biochemical analysis were done for 671 unrelated participants (obese=333 and non-obese=338) (age≥18 years). Genotyping of the polymorphisms was done by PCR-RFLP method. However, 50% of the samples were sequenced by Sanger sequencing.

**Results:**

Both the *FTO* variants 30685 (TT vs GG: odds ratio (OR), 2.30; 95% confidence interval (CI), 1.39-3.79) and -23525 (TT vs AA: odds ratio (OR), 2.78; 95% confidence interval (CI), 1.37-5.64) showed substantial risk towards obesity by conferring it 2 times and 3 times, respectively. The analysis by logistic regression showed a significant association for both the variants 30685T/G (rs17817449) and -23525T/A (rs9939609) (OR=2.29; 95%CI: 1.47-3.57) and (OR=5.25; 95% CI: 2.68-10.28) under the recessive genetic model, respectively. The haplotype combination TA (30685; -23525) develops a 4 times risk for obesity (*P*=0.0001). Among obese, the G allele of 30685T/G and A- allele of -23525T/A showed variance in Body mass index (BMI), waist circumference (WC), waist-to-height ratio(WHtR), systolic blood pressure (SBP), diastolic blood pressure (DBP) and triglyceride(TG).

**Conclusion:**

The present investigation indicated that both the *FTO* 30685T/G (rs17817449) and -23525T/A (rs9939609) polymorphisms have a key impact on an individual’s vulnerability to obesity in this population.

## Introduction

 Obesity is a common multifactorial disorder characterized by the accretion of excess body fat. Obesity has been increasing at an alarming rate in low- and middle-income countries during the last three decades.^1^ Obesity and overweight are the main risk factors for many non-communicable diseases considering 44% of diabetes, 23% of ischaemic heart disease and 7-41% of certain cancers.^2,3^ Various factors contribute to obesity like binge eating, a sedentary lifestyle, lack of physical activity, alcohol consumption, and consumption of a high-calorie-rich diet. BMI, or Body Mass Index, which is calculated as the ratio of Weight in Kg to height in m^2^ is a globally employed measure for evaluating overweight and obesity.

 According to WHO, individuals with a body mass index (BMI) greater than 25 kg/m^2^ are considered overweight, while those with a BMI exceeding 30 kg/m^2^ are classified as obese.^4^ However, it has always been contentious for Asian and South Asian populations. Despite having a lower BMI of 25 kg/m^2^, they exhibit a high incidence of cardiovascular disease, abdominal obesity, trunk subcutaneous fat and a notable difference in percent body fat. This challenges the conventional BMI cutoffs.^5,6^ The recommended BMI cut-off points for the Asian population were as follows: the normal range is 18.5-22.9, overweight is defined as > 23.0 to 24.9, obese class I is ≥ 25.0 to 29.9, and obese class II is ≥ 30.0.^7^ It has been found that by the year 2040, the prevalence of overweight among adults in India is anticipated to undergo a twofold escalation, while the incidence of obesity is expected to undergo a threefold amplification.^8^ Numerous genes contribute to obesity predisposition, with various single nucleotide polymorphisms (SNPs) in these genes linked to the disease across diverse populations. However, these positive associations are not found in every population possibly due to differences in various factors like lifestyle, ethnic background, eating behaviour etc.^9-13^

 The *FTO* (Fat mass and obesity-associated) gene is present on chromosome no. 16q12.2 and comprises 9 exon counts. This gene spans about 417,979 bp. The variants of the *FTO* SNPs are present in 47 kb linkage disequilibrium blocks covering the portion of 1^st^ two introns and exon 2 of the *FTO* gene that is related to obesity and high body mass index. The *FTO* gene cipher a Fe II 2-oxoglutarate dependent demethylase enzyme that cuts the methyl group present in various positions of DNA as well as RNA and it is expressed in the arcuate nucleus of the hypothalamus playing a key role in the mechanism of energy intake and energy expenditure.^14^ Several studies have examined a remarkable correlation between obesity risk and polymorphisms of the *FTO* gene.^15-19^ The current study depicts two intronic variants of the *FTO* gene 30685T/G (rs17817449) and -23525T/A (rs9939609).

 An intronic variant 30685T/G substitution shows a significant relation with obesity and its related phenotypes.^16,20-24^ The *FTO* 30685T/G variant is linked to the consumption of high-fat-rich foods and refined starches.^25^ Moreover, an association has been observed with increased triglyceride levels for the 30685T/G variant.^26^ Another intronic variant in the *FTO* gene having T to A substitution (-23525T/A) shows an association with high BMI in obese individuals.^26-29^ Several studies have explored the association between the *FTO* gene polymorphism and metabolic syndrome, revealing contradictory findings. While some studies have established a connection, others have not found any association between *FTO* gene polymorphisms and obesity or overweight.

 Obesity is influenced by genetic variations and ethnicity, with known polymorphisms showing varied associations across different ethnic populations. The population of Punjab, India has a unique genetic profile and extensive diversity. The study aims to assess the connection between SNPs in the *FTO* gene and obesity susceptibility in this population due to the high prevalence of overweight and obesity in the region.

## Materials and Methods

###  Study Subjects

 The present population-based case-control study included 671 unrelated adult individuals recruited from different districts of Punjab, an Indian state. Out of 671 subjects 333 were obese caseswith a mean age of 39.65 ± 10.25 years and a mean BMI of 27.57 ± 1.37 kg/m^2^ for obese I, and 34.03 ± 4.28 kg/m^2^ for obese II.Whereas, 338 were non-obese healthy controlswith a mean age of 37.46 ± 11.56 years and a mean BMI of 21.01 ± 1.89 kg/m^2^. To minimize the selection bias in the present case-control study, the controls were randomly collected from the same population with matching ages and a 1:1 ratio to improve the statistical power of the study. The inclusion criteria for obese cases were adult individuals (≥ 18 years) with a BMI exceeding 25 kg/m^2^, categorized as Obese I (25-29.9 kg/m^2^) and Obese II (≥ 30 kg/m^2^). Controls were individuals with a BMI ranging from ≥ 18.5 to 22.9.^7^ The exclusion criteria were pregnant and lactating females, individuals with big muscles, diabetes, kidney disease and other cardiovascular disease. All the subjects were interviewed and their ethnographic information, drug intake, anthropometric measurements, socio-economic lifestyle patterns, and family history were noted down.

 The present study protocol was according to the Declaration of Helsinki (1964). All participants submitted written consent before their inclusion in this study and the study was agreed upon by the Institutional ethics committee of Guru Nanak Dev University.

###  Sample size calculation

 The determination of the sample size for the present case-control study was conducted using the CaTS power software, specifically designed for genetic association studies.^30^ The calculation of the sample size involved following specific assumptions. Firstly, a two-stage sample design was employed. The accepted significance level, denoted as Type II error, was set at 0.05. The prevalence rate of obesity in the Punjab region was considered25% according to Tripathy et al^31^ To identify an association with an effective size, the odds ratio of genotype-related risk was established at 1.50. Additionally, an additive model was utilized to ensure a statistical power exceeding 90%, along with a 95% confidence interval. Based on these calculations, the final sample size arrived at 333 obese cases and 338 healthy controls by increasing it by 10% to account for statistical correction, adjustment/design effect, and improved statistical power and level of significance for both cases and controls.

###  Anthropometric and Physiometric measurements

 Various parameters like height, weight, waist circumference (WC), hip circumference (HC), and skinfold measurements were noted. The height of the subject was measured to the nearest 0.5 cm whereas the weight of the subject was measured to the nearest 0.1 kg and these measurements were taken with bare feet and light clothing. Waist, arm, calf and hip circumference were measured in cm. BMI was calculated by the given formula- the ratio of weight in kg to height in m^2^. WHR was calculated by the ratio of waist circumference to the hip circumference whereas, WHtR was calculated by the ratio of waist circumference to that of height. Skinfold measurements of triceps, bicep, supra-iliac and sub-scapular were taken in mm by Lange skinfold caliper. For physiometric measurements such as systolic and diastolic blood pressures were measured by a mercury sphygmomanometer. Mean arterial blood pressure (MBP) was also calculatedby the given formula^32^:

 MBP = Diastolic Blood Pressure (DBP) + 1/3 [Systolic Blood Pressure (SBP)- DBP]

###  Biochemical Analysis

 Serum was obtained from venous blood samples after an overnight fast to measure lipid variables, including total cholesterol (TC), triglyceride (TG) and high-density lipoprotein (HDL) by using Erba- Mannheim kit (Trans Sasia Bio-medicals Ltd., Solan, India). The calculation of low-density lipoprotein (LDL) and very low-density lipoprotein (VLDL) was done by Friedewald formula.^33^

###  Genetic analysis

 Genomic DNA was isolated by the Phenol/chloroform (PCA) method which was then diluted to a working concentration of 50ng/ml and stored at -20ºc for further use. Genotyping for both polymorphisms of the *FTO* gene was done by Polymerase chain reaction-based Restriction fragment length polymorphism (PCR-RFLP) and the Sanger sequencing method. The DNA was amplified by following set of primers: F: 5’-CGGTGAAGAGGAGGAGATTG-3’ and R: 5’-CATCTCTGCCCCAGTTTCTC-3’ for 30685T/G; F: 5’- AACTGGCTCTTGAATGAAATAGGATTCAGA-3’ and R: 5’- AGAGTAACAGAGACTATCCAAGTGCAGT AC-3’ for -23525T/A.^34,35^

 The PCR was performed by taking 50ng of DNA, 200µM of each dNTP, 4µM of each primer, 1x PCR buffer and 0.3U/ml of Taq DNA polymerase in a total reaction volume of 15µl. The conditions of the PCR cycle were: initial denaturation of 5 min at 95ºc followed by 30 cycles of denaturation for 45sec at 95ºc, annealing of 1min at 58ºc (for 30685T/G), 30 sec at 58ºc (for -23525T/A) extension of 1 min at 72ºc and a final extension of 10 min at 72ºc. To investigate contamination in PCR, a negative control without a DNA template was run with each reaction. The PCR products were checked in 2% agarose gel stained with ethidium bromide (10mg/ml) (Genei^TM^).

 The amplified PCR products (223bp for 30685T/G and 182bp for -23525T/A) were digested with restriction enzyme AlwNI and ScaI (New England Biolabs, USA) for both the polymorphisms 30685T/G and -23525T/A, respectively for 12-16 hours at 37ºc with 1x Neb buffer in a final reaction volume of 15µl followed by heat inactivation at 65ºc for 20min. The digested PCR products were resolved in 2.5% agarose gel stained with ethidium bromide against a 100 bp ladder and viewed under a UV transilluminator.

**Figure 1:**
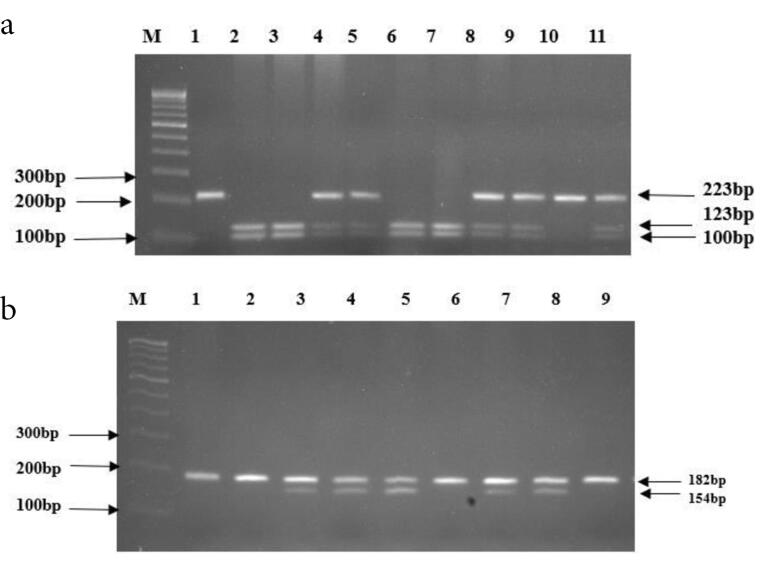
Ethidium bromide-stained 2.5% agarose gel pictures showing PCR-RFLP digested products of the *FTO* gene polymorphisms (a) 30685T/G; M: 100bp DNA ladder, Lane 2,3,6,7 represents wild genotype (TT), Lane 1, 10 represents mutant genotype (GG), Lane 4,5,8,9,11 represents heterozygous genotype (TG) (b) -23525T/A; M: 100bp DNA ladder, Lane 1,2,6,9 represents wild genotype (TT), Lane 3,4,5,7,8 represents heterozygous genotype (TA)

 In 30685T/G, the homozygous wildtype allele was digested and resulted in 2 fragments of 123 bp and 100 bp i.e., the mutant allele remains undigested (Figure 1a). On the contrary, in -23525T/A the homozygous wildtype allele remains undigested whereas, the mutant allele gave 2 fragments of 154 bp and 28bp on digestion (Figure 1b).

###  Sequencing

**Figure 2:**
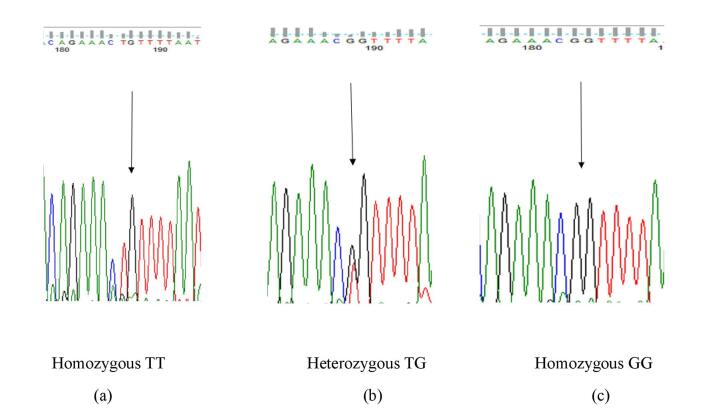
Sequencing chromatograms demonstrating the genotypes for polymorphism 30685T/G (a) Homozygous TT (b) Heterozygous TG (c) Homozygous GG

**Figure 3:**
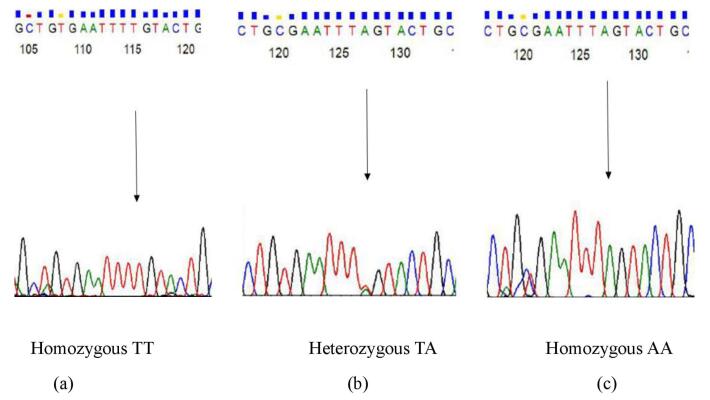
Sequencing chromatograms demonstrating the genotypes for polymorphism 30685T/G (a) Homozygous TT (b) Heterozygous TA (c) Homozygous AA

 Genotyping of 50% of the samples for each polymorphism was performed by the Sanger sequencing method. Figures 2 and 3 demonstrate the sequencing chromatograms for the genotypes of 30685T/G and -23525T/A polymorphism. Individual sequence traces were examined, and alignments were compared to GenBank sequences using the NCBI blast program.

###  Statistical analysis

 The data were analyzed using Statistical Package for Social Sciences (SPSS) for Windows Version 21.0. Continuous variables were represented as means ± SD and were compared by the student’s t-test, and quantitative data of the three groups were compared by one-way ANOVA. Binary logistic regression was performed to test the association of studied variants, under presumed genetic models after adjusting for various confounding factors. Allelic and genotypic frequencies were compared between obese cases and non-obese controls using the chi-square test (χ^2^). The influence of clinical variables and polymorphisms in the risk of obesity was examined with multiple linear regression analysis. To assess the effect of *FTO* gene variants on traits defining obesity, linear Regression analysis was performed.

 The construction of a linkage disequilibrium map and haplotype blocks within variants of the *FTO *gene was based on genotypes and utilized Haploview software (version 4.1). Haplotype frequencies, odds ratios (ORs), two-tailed p-values and 95% confidence interval (CI) were calculated as the measure of association of the SNPs and the presence of obesity. A two-tailed p-value < 0.05 was considered statistical standard significant.

## Results

###  Clinical and Biochemical characteristics of the study population

Table 1 summarizes the comparison of various anthropometric, clinical, and biochemical data among the obese I, obese II and control groups. The statistical analysis revealed that the mean values for height, weight, hip circumference (HC), waist circumference (WC), calf circumference (CC), arm circumference (AC), body mass index (BMI), triceps skinfold (TSF), biceps skinfold (BSF), subscapular skinfold (SSSF), suprailiac skinfold (SISF), waist-to-height ratio (WHtR), systolic blood pressure (SBP), pulse pressure (PP), total cholesterol (TC), triglyceride (TG), and low-density lipoprotein (LDL) were significantly higher in both obese I and obese II groups compared to the non-obese group (*P* < 0.001).

**Table 1:** Anthropometric, clinical and biochemical characteristics of obese and non- obese

**Variable**	**Non obese** **(n=338)**	**Obese I (n=145)**	**Obese II (n=188)**	**p- value**
Height (cm)	161.53 ± 8.50	162.63 ± 10.02	159.46 ± 10.29	**0.00743**
Weight (kg)	54.90 ± 7.41	73.07 ± 9.68	86.60 ± 12.40	**0.00001**
BMI (kg/m^2^)	21.01 ± 1.89	27.57 ± 1.37	34.03 ± 4.28	**0.00001**
WC (cm)	80.26 ± 10.59	92.21 ± 9.77	109.99 ± 17.88	**0.00001**
HC (cm)	91.60 ± 7.27	101.02 ± 10.06	124.38 ± 23.43	**0.00001**
AC (cm)	25.35 ± 6.80	27.84 ± 4.13	31.58 ± 4.52	**0.00001**
CC (cm)	29.86 ± 4.07	33.39 ± 3.95	35.71 ± 5.18	**0.00001**
WHR	0.87 ± 0.07	1.30 ± 3.47	1.01 ± 0.23	0.20125
WHtR	0.49 ± 0.65	0.56 ± 0.05	0.68 ± 0.11	**0.00001**
TSF	16.95 ± 7.53	20.90 ± 9.22	25.77 ± 8.80	**0.00001**
BSF	12.81 ± 7.80	15.79 ± 9.24	21.95 ± 9.94	**0.00001**
SSSF	19.17 ± 8.54	23.63 ± 9.11	29.73 ± 9.14	**0.00001**
SISF	18.88 ± 10.18	23.42 ± 8.66	28.28 ± 9.53	**0.00001**
SBP (mmHg)	119.69 ± 21.93	122.64 ± 8.10	127.16 ± 17.04	**0.00201**
DBP (mmHg)	79.55 ± 11.26	79.83 ± 10.64	81.23 ± 11.19	0.32609
MBP (mmHg)	93.08 ± 14.34	96.69 ± 16.06	96.34 ± 11.96	0.087026
PP	40.05 ± 14.82	42.72 ± 14.08	46.15 ± 15.35	**0.00185**
PR	77.36 ± 10.18	78.37 ± 10.31	77.57 ± 9.19	0.64614
TC (mg/dl)	163.73 ± 21.80	163.94 ± 23.71	207.88 ± 44.44	**0.00001**
TG (mg/dl)	65.83 ± 25.86	68.09 ± 31.63	161.64 ± 37.94	**0.00005**
HDL (mg/dl)	46.91 ± 13.86	48.42 ± 17.24	54.93 ± 24.08	0.24270
VLDL (mg/dl)	18.76 ± 26.47	13.61 ± 7.92	32.32 ± 21.38	0.10991
LDL (mg/dl)	94.66 ± 35.44	101.84 ± 35.61	118.16 ± 33.55	**0.00394**

Mean ± standard deviation.Obese I: BMI 25-29.9kg/m^2^; Obese II: BMI ≥ 30kg/m^2^ CC, calf circumference; WC, waist circumference; WHR, waist to hip ratio; AC, arm circumference; WHtR, waist to height ratio; sub-scapular skinfold; BSF, bicep skinfold; SSSF, TSF, triceps skinfold; SISF, supra-iliac skinfold; HC, hip circumference SBP, systolic blood pressure; MBP, mean blood pressure; BMI, body mass index; DBP, diastolic blood pressure; PP, pulse pressure; TC, total cholesterol; LDL, low density lipoprotein; TG, triglyceride; HDL, high density lipoprotein; PR, pulse rate; VLDL, very low density lipoprotein. Statistically significant p values are represented in bold derived from one-way ANOVA analysis.

###  Association between genotypes with clinical parameters

 The analysis of clinical and biochemical parameters across the genotypes among both studied polymorphisms has been presented in Table 2. The risk genotypes of both the variants i.e., 30685GG and -23525AA genotypes had significantly higher BMI (*P* = 0.00001 and *P* = 0.00001); WC (*P* = 0.00450 and 0.00358); SBP (*P* = 0.00003 and 0.0002); diastolic blood pressure DBP (*P* = 0.00001 and 0.0019); TC (*P* = 0.01478 and 0.02598) and TG (*P* = 0.03346 and 0.00005), respectively. However, the high-density lipoprotein HDL (*P* = 0.00145 and 0.04254) was found to be significantly higher in individuals harboring 30685TT and -23525TT genotypes respectively. Furthermore, the genetic variant 30685T/G demonstrated a significant association with HC, WHR, BSF, SISF and pulse rate (PR) (*P* < 0.05). Similarly, the variant -23525T/A showed significant associations with weight, very low-density lipoprotein (VLDL) andLDL (*P* < 0.05).

**Table 2:** Clinical characteristics of the study population according to *FTO* 30685T/G and -23525T/A genotypes

**SNPs**	**30685 T/G**			**-23525T/A**			**p-value**
**Parameters**	**TT**	**TG**	**GG**	**TT**	**TA**	**AA**	
Height (cm)	160.54 ± 8.48	162.37 ± 8.33	158.02 ± 6.41	162.74 ± 7.40	158.76 ± 6.55	156.31 ± 6.20	**0.00006**^a^ **0.00001**^b^
Weight (Kg)	70.45 ± 12.82	71.04 ± 10.93	72.98 ± 11.37	69.79 ± 8.95	70.62 ± 10.11	64.49 ± 6.76	0.22886^a^ **0.00006**^b^
BMI (Kg/m^2^)	23.05 ± 2.45	25.95 ± 3.59	27.65 ± 4.05	24.84 ± 4.01	28.85 ± 5.59	29.92 ± 5.98	**0.00001**^a^ **0.00001**^b^
WC (cm)	92.79 ± 14.29	96.95 ± 13.96	98.08 ± 16.32	93.70 ± 12.75	95.03 ± 13.64	100.41 ± 12.13	**0.00450**^a^ **0.00358**^b^
HC (cm)	102.94 ± 14.52	105.72 ± 13.75	107.99 ± 18.49	103.18 ± 12.04	104.94 ± 13.32	106.73 ± 13.75	**0.02567**^a^ 0.13877^b^
WHR	0.89 ± 0.08	0.95 ± 0.17	0.97 ± 0.16	0.94 ± 0.16	0.95 ± 0.17	0.98 ± 0.19	**0.00001**^a^ 0.34651^b^
BSF (mm)	19.74 ± 6.29	19.47 ± 6.96	21.56 ± 4.28	18.20 ± 7.26	19.08 ± 8.69	18.02 ± 7.63	**0.02181**^a^ 0.45228^b^
SISF (mm)	23.12 ± 7.13	25.42 ± 8.98	25.38 ± 6.73	23.58 ± 7.90	23.72 ± 8.26	23.86 ± 6.87	**0.01067**^a^ 0.96916^b^
SBP (mmHg)	123.34 ± 17.98	123.64 ± 17.30	132.69 ± 16.86	124.22 ± 16.70	123.91 ± 16.99	133.78 ± 13.18	**0.00003**^a^ **0.00020**^b^
DBP (mmHg)	79.27 ± 10.27	81.94 ± 10.53	86.54 ± 9.48	80.35 ± 10.56	79.59 ± 10.39	85.04 ± 8.70	**0.00001**^a^ **0.00190**^b^
PR	76.67 ± 8.88	78.05 ± 9.67	81.39 ± 8.91	76.14 ± 7.68	77.49 ± 9.19	78.26 ± 8.61	**0.00035**^a^ 0.14130^b^
TC (mg/dl)	163.27 ± 23.14	173.41 ± 22.57	186.15 ± 29.42	178.03 ± 21.24	168.12 ± 18.17	183 ± 18.28	**0.01478**^a^ **0.02598**^b^
TG (mg/dl)	147.32 ± 33.91	155.84 ± 35.79	174.52 ± 26.63	135.35 ± 25.87	124.46 ± 24.75	169.33 ± 17.22	**0.03346**^a^ **0.00005**^b^
HDL (mg/dl)	50.57 ± 14.62	44.01 ± 7.86	37.91 ± 6.31	52.98 ± 12.49	48.43 ± 21.71	38.20 ± 8.20	**0.00145**^a^ **0.04254**^b^
VLDL (mg/dl)	18.80 ± 7.47	24.05 ± 19.50	20.08 ± 18.19	30.72 ± 21.11	21.74 ± 14.30	52.30 ± 16.09	0.47182^a^ **0.00009**^b^
LDL (mg/dl)	117.28 ± 26.62	124.35 ± 18.82	132.04 ± 24.24	123.28 ± 21.60	109.68 ± 38.35	136.48 ± 23.49	0.13518^a^ **0.03431**^b^

^a^Comparison for 30685T/G ^b^Comparison for -23525T/A Statistically significant p values are represented in bold derived from one-way ANOVA analysis.

###  Distribution of FTO genotypes and alleles in obese and non-obese groups

 The genotype and allele frequency distributions for *FTO *30685T/G(rs17817449) and -23525T/A(rs9939609) polymorphisms in the total study population, obese and non-obese groups are presented in Table 3. The overall genotype (*P* = 0.00001; 30685T/G and *P* = 0.0005; -23525T/A, respectively) and allele frequencies (*P* = 0.0063; 30685T/G and *P* = 0.0001;-23525T/A) differed significantly between the obese and non-obese groups for both polymorphisms. The homozygous recessive genotype 30685GG was more prevalent in the obese group (20.42%) compared to the non-obese group (10%); (*P* = 0.0001). The 30685GG exhibited a twofold higher risk for the development of obesity [OR(95%CI): 2.30 (1.39-3.79); *P* = 0.001]. Similarly, the frequency of the homozygous recessive genotype -23525AA was significantly higher in the obese group (15.01%) compared to the non-obese group (3.25%) (*P* = 0.0001). The -23525AA genotype conferred an almost threefold higher risk for the progression of obesity [OR (95%CI): 2.78 (1.37-5.64); *P* = 0.0046].

**Table 3:** Genotype and allele frequency distribution of *FTO* gene polymorphisms in obese and non-obese individuals

**Polymorphism**	**Genotypes/** **Alleles**	**Total study population (671) %(n)**	**Obese (333) %(n)**	**Non-obese (338) %(n)**	**OR (95% CI)**	**p**^a^**-value**	**p**^b^**-value**	**p**^c^**-value**
30685T/G	TT	238 (37.6)	118 (35.44)	135 (40)	**Reference**	-	0.249	
	TG	297 (46.92)	147 (44.14)	169 (50)	0.99(0.71-1.40)	0.984	0.431	**0.0001**
	GG	98 (15.48)	68 (20.42)	34 (10)	2.30(1.399-3.79)	**0.001**	**0.0001**	
	T allele	773 (61.06)	383 (57.50)	439 (65)	**Reference**	-		**0.0063**
	G allele	493 (38.94)	283 (42.50)	237 (35)	1.37(1.09-1.72)	**0.0064**		
-23525T/A	TT	316 (47.1)	134 (40.24)	182 (53.85)	**Reference**	_	**0.001**	
	TA	294 (43.81)	149 (44.75)	145 (42.9)	2.02(01.31-3.12)	**0.0014**	0.331	**0.00005**
	AA	61 (9.09)	50(15.01)	11 (3.25)	2.78(1.37-5.64)	**0.0046**	**0.0001**	
	T allele	926 (69)	417 (62.61)	509 (75.3)	**Reference**	_		**0.0001**
	A allele	416 (31)	249 (37.39)	167 (24.7)	1.82(1.43-2.30)	**0.0001**		

p^a^- values for odd ratiosderived from Binary logistic regression analysis. p^b^- comparison between obese and non- obese subjects for each genotypederived from χ^2^ test. p^c^- comparison between obese and non-obese subjects for overall genotypesderived from χ^2^ test. OR- odds ratio, CI- confidence interval. Statistically significant values are represented in bold.

###  Association of genetic models with obesity

 The results of logistic regression analysis using different genetic models to assess the association between *FTO* gene polymorphisms with obesity are shown in Table 4. The 30685T/G and -23525T/A polymorphismsconferred a huge risk for obesity by conferring it 2-fold and 5 folds under the recessivemodel of inheritance [OR (95%CI): 2.29 (1.47-3.57); *P* = 0.0007; OR (95%CI): 5.25 (2.68-10.28); *P* = 0.0001, respectively].

**Table 4:** The proposed genetic models with odds ratio for 30685T/G and -23525T/A associated with obesity

**SNP**	**Genetic Model**	**OR (95% CI)**	**p-value**
30685T/G	Dominant	**Reference**	**-**
	Recessive	**2.29(1.47-3.57)**	**0.0007**
	Co-dominant	0.995(0.714-1.386)	0.977
-23525T/A	Dominant	**Reference**	-
	Recessive	**5.25(2.68-10.28)**	**0.0001**
	Co-dominant	1.39(1.01-1.92)	0.058

Abbreviations: CI, confidence interval; OR odds ratio; SNP, single nucleotide polymorphism. *P* < 0.05 are provided in bold to emphasize their significance.p-values were derived from Binary logistic regression analysis.

###  Haplotype analysis

**Figure 4:**
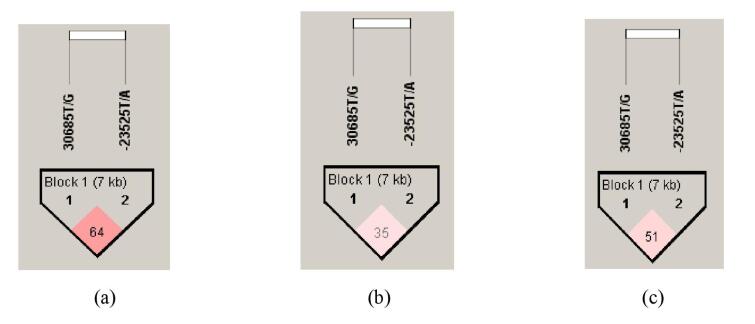
LD plot showing the position of the two *FTO* polymorphisms and pair-wise D’ values observed in the study population with respect to (a) Study population (b) Obese cases (c) non-obese controls

 The haplotype distribution and the pattern of LD in both *FTO *polymorphisms in the total study population, obese and non-obese groups are presented in Table 5. The haplotype frequencies were significantly different between obese and non-obese subjects (*P* = 0.0001). The TT haplotype (both wild-type alleles) exhibited a higher frequency in the control group, signifying a significant protective effect against obesity development [OR (95%CI): 0.64 (0.52-0.79); *P* = 0.0001]. On the contrary, the GT and TA haplotypes were more frequent in obese subjects. The high-risk GA haplotype (30685G and -23525A) was considered as the baseline haplotype. When compared to this baseline haplotype, the G-T haplotype significantly elevated the risk of obesity [OR (95%CI): 3.1 (2.20-4.36); *P* = 0.0001]. Similarly, the TA haplotype, comprised of low-risk 30685T and high-risk -23525A significantly increased the obesity risk [OR (95%CI): 4.87 (2.96-7.99); *P* = 0.0001]. Based on the measure of LD, it has been observed the two SNPs (30685T/G and -23525T/A) were in strong LD in the total study population (D’ = 0.644, r^2^ = 0.323) whereas, in moderate LD among obese and non-obese groups (D’ = 0.351, r^2^ = 0.091; D’ = 0.517, r^2^ = 0.218, respectively (Figure 4).

**Table 5:** Evaluation patterns of LD in *FTO *polymorphisms and haplotype frequencies.

**Haplotypes**		**Study- Population** **%(n) (671)**	**Obese ** **%(n) (333)**	**Non-obese** **%(n) (338)**	**OR (95CI%) **^a^	***P***^a^**-value**	***P***^b^**-value**	***P***^c^**-value**
**30685T/G**	**-23525T/A**							
G	A	28.1(188)	21.6(72)	33.2(112)	**Reference**	----	**0.001**	**0.0001**
G	T	13.3(89)	20.3(67)	7.6(26)	**3.1(2.20-4.36)**	**0.0001**	**0.001**	
T	A	7.4(51)	13.1(44)	3.0(10)	**4.87(2.96-7.99) **	**0.0001**	**0.001**	
T	T	51.2(343)	45(150)	56.2(190)	**0.64(0.52-0.79) **	**0.0001**	**0.005**	
**LD measure**		**Study population**		**Obese cases**			**Non –obese controls**	
		**D’**	**r**^2^	**D’**	**r**^2^		**D’**	**r**^2^
30685T/G and -23525T/A		0.644	0.323	0.351	0.091		0.517	0.218

Statistically significant values are represented in boldderived from χ^2^ test. ^a^*p* values and OR values derived from comparing each haplotype with the baseline haplotype (G-A). ^b^*p* values derived from comparing a specific haplotype with the other three. ^c^*p* Comparison between obese and non-obese subjects for overall haplotypes.

###  Interaction analysis of FTO 30685T/G and -23525T/A polymorphism

 The interaction analysis of different combinations of two polymorphisms has been presented in Table 6. The combination of dominant homozygous (TT) of 30685T/G and risk genotype (AA) of -23525T/A [OR (95%CI): 0.16 (0.05-0.49); *P* = 0.0004]; heterozygous TG of 30685T/G and risk genotype (AA) of -23525T/A [OR (95%CI): 0.19 (0.07-0.51); *P* = 0.0004]; risk genotype (GG) of 30685T/G and dominant homozygous (TT) of -23525T/A [OR (95%CI): 0.48 (0.24-0.95); *P* = 0.033]; risk genotype (GG) of 30685T/G and heterozygous (TA) of -23525T/A [OR (95%CI): 0.33 (0.17-0.69); *P* = 0.003] have exhibited protection against the development of obesity, respectively.

**Table 6:** Frequency distribution of combination genotypes in 30685T/G and -23525T/A and interaction analysis of various genotype combinations.

**Genotype Combination** **30685T/G -23525T/A**	**Obese cases** **n(%)**	**Non-obese** **controls n(%)**	**χ**^2^	**OR (95% CI)**	**p-value**
TT-TT	66(19.76)	91(26.92)	**Reference**	---	---
TT-TA	53(15.86)	58(17.16)	0.86	0.79(1.15-1.29)	0.383
TT-AA	18(5.32)	4(1.30)	12.26	0.16(0.05-0.49)	**0.0004**
TG-TT	59(17.77)	73(21.54)	0.21	0.89(0.56-1.43)	0.720
TG-TA	48(14.26)	66(19.45)	2.0	0.99(0.61-1.6)	0.92
TG-AA	22(6.62)	6(1.62)	12.72	0.19(0.07-0.51)	**0.0004**
GG-TT	27(8.21)	18(5.38)	4.54	0.48(0.24-0.95)	**0.033**
GG-TA	30(9.13)	14(4.30)	9.41	0.33(0.17-0.69)	**0.003**
GG-AA	10(3.07)	8(2.33)	1.2	0.58(0.22-1.55)	0.32

Interactive association was assessed between the genotype statuses of the studied polymorphisms by 2 × 2 chi square contingency table. p-value is significant at > 0.05 levelderived from χ^2^ test.

###  Association of FTO polymorphisms with obesity indices

 Multiple linear regression analysis was performed to investigate the impact of 30685T/G (rs17817449) and -23525T/A (rs9939609) polymorphisms in elucidating the variation in obesity-related traits Table 7. The results revealed that the 30685G allele contributed with 29.59 (*P* = 0.0001), -0.188 (*P* = 0.013), 4.22 (*P* = 0.01), 13.45 (*P* = 0.012), -0.083 (*P* = 0.002), -275 (*P* = 0.035) and 0.02 (*P* = 0.001) increase in BMI, WC, WHR, waist to height ratio (WHtR), SBP, DBP and TG concentration, respectively. Furthermore, -23525A contributed with 20.13 (*P* = 0.0001), 0.031 (*P* = 0.030), 6.62 (*P* = 0.004), -0.014 (*P* = 0.0001), 0.02 (*P* = 0.009) and 0.007 (*P* = 0.0001) increase in BMI, WC, WHtR, SBP, DBP and TG, respectively. The 30685T/G polymorphism and the selected covariates were shown to be significant and independent predictors of the BMI, WC, WHR, WHtR, SBP, DBP and TG among obese subjects explaining the 55.5%, 49.1%, 13.3%, 42.3%, 34.7%, 31.1% and 22.3% of the variance, respectively. Similarly, the -23525T/A polymorphism also contributed 98%, 82.1%, 54%, 59.1%, 71.8% and 84.7% of the variability in the BMI, WC, WHtR, SBP, DBP and TG, respectively.

**Table 7:** Multiple linear regression analysis: influence of FTO polymorphisms on variables associated with obesity

**Variables **	**30685T/G**				**-23525T/A**			
	**B(SE)**	**r**	**P-value**	**R**^2^**(%)**	**B(SE)**	**r**	**P-value**	**R**^2^**(%)**
BMI	29.59(4.88)	0.678	**0.0001**	55.5	20.137(0.375)	0.99	**0.0001**	98
WC	-0.188(0.072)	0.536	**0.013**	49.1	0.031(0.014)	0.906	**0.030**	82.1
WHR	4.22(1.47)	0.321	**0.01**	13.3	-0.200(0.638)	0.067	0.755	0.2
WHtR	13.45(5.155)	0.461	**0.012**	42.3	6.62(2.13)	0.736	**0.004**	54
SBP	-0.083(0.025)	0.375	**0.002**	34.7	-0.014(0.004)	0.769	**0.0001**	59.1
DBP	-275(0.041)	0.103	**0.035**	31.1	0.020(0.007)	0.848	**0.009**	71.8
TC	0.007(0.006)	0.139	0.385	14.4	0.001(0.001)	0.878	0.339	76.6
TG	0.02(0.006)	0.472	**0.001**	22.3	0.007(0.0001)	0.922	**0.0001**	84.7

r: partial regression coefficient, R^2^: coefficient of determinant

## Discussion

 We designed this case-control study to evaluate the association of two* FTO* variants 30685T/G (rs17817449) and -23525T/A (rs9939609) with obesity risk in the population of Punjab. Obesity is a complex phenotype and is contributed by genetic and environmental factors.^3,6^ Among the genetic factors, the *FTO* gene has been considered one of the primary contributors to the development of the risk of polygenic obesity. Despite this fact, the impact of *FTO* variants has been a subject of controversy among oceanic, Egyptian, Portuguese, Iranian, Brazilian and other multi-ethnic populations.^9,12,13,26,36,37^ The present investigation has reported a significant association of 30685GG and -23525AA genotypes with the risk of obesity and its related parameters in the studied population. Individuals carrying 30685GG and -23525AA genotypes have twice more risk for developing obesity than non-carriers suggesting that *FTO *30685GG and -23525AA might be a good predictor for obesity in this population.

 The present findings align with numerous previous studies conducted among various populations, including Saudi, Thai, Spanish, Indian, South African and Mexican, confirming the interaction of these two polymorphisms with obesity.^16,21,38-41^ However, the present results contrast with numerous previous studies conducted in oceanic, Chinese, Egyptian, Mexican and Latin American populations.^9,10,12,35,42^ The possible reason for these conflicting results could be that *FTO* gene products play a role in regulating food intake, with individuals carrying the risk allele tending to opt for food with higher energy content and increased fat.^43^ In the Egyptian population, the two *FTO* gene polymorphisms exhibited no association with obesity. This could be attributed to the significant role of ethnicity variations in influencing the genetic component of individuals and their susceptibility to obesity.^12^ Li et al reported significant differences in *FTO *risk allele frequencies and gene linkage patterns among the Chinese population, with risk alleles being common in Europeans but not in the Chinese.^10^ These differences suggest an evolutionary divergence that might reflect a history of negative selection against the *FTO *risk alleles in the Chinese population. Additionally, the potential variance in the genetic architecture of different ethnic groups may contribute to the distinct actions of these SNPs in different populations. Similarly, a study conducted on an oceanic population has elucidated that the *FTO* gene experienced negative selection following the divergence of mouse and human lineages.^9^ A study on Latin Americans revealed that communities have experienced a substantial nutrition transition, where socioeconomic factors such as urbanization and income were likely major contributors to the significant interpersonal variability in BMI. This suggests that the impact of *FTO* genetic polymorphisms on obesity susceptibility might be mitigated by socioeconomic variables.^42^

 The specific biological process through which the *FTO* polymorphisms contribute to the heightened susceptibility to obesity remains largely unidentified.^44^ Clarifying how the *FTO* gene polymorphisms influence fat mass could enhance our comprehension of the origins of obesity. The *FTO* primarily contributes to weight gain by elevating energy intake and reducing the sensation of satiety. The* FTO* gene’s intronic region, highly sensitive to DNase, binds various transcription factors associated with obesity, particularly showing robust signals with glucocorticoid receptors.^45^ *FTO* gene polymorphisms impact RNA-level gene regulation through catalytic demethylation leading to the development of obesity, ultimately contributing to insulin resistance.^14^

 Studies suggest that plasma adiponectin and satiety hormones like leptin, released from adipose tissue, are affected by*FTO* gene polymorphisms.^46^ These genetic variants of the *FTO* gene are responsible for reducing post-prandial satiety consequently increasing hunger.^46^ Similarly, adiponectin is vital for glucose uptake and fatty acid metabolism. Although the mechanism explaining the potential connection between *FTO* and adiponectin is not clearly defined, individuals with obesity or elevated BMI often show reduced levels of adiponectin.^47^

 Adipose tissue also produces high-sensitive C-reactive protein (hsCRP), recognized as a pro-inflammatory cytokine linked to obesity-related inflammation. The *FTO *gene polymorphisms contribute not only to increased adiposity but also potentially amplify inflammation within adipose tissue, leading to heightened systemic inflammation regardless of adiposity levels.^48^ Additionally, CRP binds with leptin, hindering its signalling and diminishing its physiological effects.^49^

 The present study reported that both *FTO *variants were in moderate linkage disequilibrium and were associated with obesity in the Punjabi population. The present study has also suggested that common forms of obesity could be explained by the combined effects of variants located in the same gene or different genes than a single isolated variant. We also tested the influence of these polymorphisms on anthropometric and biochemical parameters in the present samples. The results revealed that 30685T/G (rs17817449) and -23525T/A (rs9939609) variants may increase the susceptibility to adiposity metabolic syndrome with increased total cholesterol (TC), waist circumference (WC), systolic blood pressure (SBP), diastolic blood pressure (DBP), body mass index (BMI), and triglyceride (TG). These results confirm the findings from other investigations in various ethnic groups and populations, including Mexican, Indian, Chinese, Brazilian, South African, Pakistani, Kuwaiti, Turkish and Italian.^15,20,21,26,39,50-53^

 The present results suggested that* FTO* 30685T/G (rs17817449) and -23525T/A (rs9939609) are the risk factors for obesity with regulation of body mass and composition. However, these two polymorphisms are not situated in an encoding region, still, they may exert functional effects through altered levels of* FTO *mRNA or in linkage disequilibrium with another genetic variant.^54^ In addition to that it has been observed that the variants located in non-coding sequences (intron1 and 2) within the *FTO *geneinteract with the promoter region of another gene *IRX3 *inthe neighborhood. Therefore, *FTO *polymorphisms association with obesity alters the expression of the *IRX3* gene in the human brain which is closely related to the regulation of obesity and its risk factors.^55^ However, more functional studies are required to confirm the role of *FTO* protein in obesity which is not completely elucidated whereas, the animal studies suggested that *FTO* expression is regulated by fasting and feeding habits.^54^

 Multiple linear regressions revealed 55%, 49%, 42%, 34%, 31% and 22% variance in BMI, WC, WHtR, SBP, DBP and TG levels respectively due to the 30685T/G variant. In relation to -23525T/A polymorphism, the results have shown statistically significant association with BMI, WC, TG, TC, DBP, SBP and WHtR accounting for 98%, 82%, 84%, 76%, 71%, 59% and 54% variability respectively. It indicates that these two variants are relevant markers for adiposity and its related metabolic indices in this population.

 The robustness of this study stems from the meticulous sampling design of our participants, who form a distinct and uniform population in terms of geographical, dietary, and cultural factors. This design is resilient to the influence of population stratification, substantially reducing the potential for false positive associations. The present study marks the initial attempt to examine the correlation between these two genetic variants and the prevalence of obesity in this particular population of Punjab. These results hold significance as the prevalence of overweight and obesity is on a rapid rise in the Indian population especially in the state of Punjab. Therefore, identifying and comprehending the mechanisms underlying the connection between the *FTO* gene and obesity will aid in developing rational strategies for personalized management of obesity.

 The present study’s sample size, particularly after BMI stratification, was insufficient for conclusive results. Therefore, it is imperative to replicate these findings in future studies with larger samples for validation.Serum levels of *FTO *were not measured in this study, preventing the conduct of genotype-phenotype correlation studies.The study sample lacks national representativeness, with significant implications mainly applicable to North Indian ethnic groups. As a result, the generalizability of these findings to other ethnicities remains uncertain. Hence, further assessments of diverse ethnic groups are necessary.

## Conclusion

 In this study, we tried to find out the impact of *FTO* variants 30685T/G (rs17817449) and -23525T/A (rs9939609) on the development of obesity risk. The results have suggested a strong association between *FTO* 30685T/G and -23525T/A polymorphisms with obesity and related phenotypes in the studied population.

## Acknowledgments

 We are highly acknowledged to all the participants for their involvement, cooperation and contribution towards this research study. The current study was assisted by departmental financial assistance from Guru Nanak Dev University, Amritsar, Punjab, India.

## Competing Interests

 The authors declare no conflict of interest.

## Ethical Approval

 The present study protocol was performed according to the Declaration of Helsinki (1964). The current study was also agreed upon by the institutional ethics committee(ethics no.1609/HG)constituted by Guru Nanak Dev University, Amritsar, Punjab, India.
